# The UK Food Environment: A Systematic Review of Domains, Methodologies, and Outcomes

**DOI:** 10.1016/j.cdnut.2025.107573

**Published:** 2025-10-11

**Authors:** Deksha Kapoor, Kirsteen Shields, Christian Reynolds, Martín Del Valle Menendez, Lindsay M Jaacks

**Affiliations:** 1 Division of Global Agriculture and Food System, University of Edinburgh, Midlothian, Edinburgh, United Kingdom; 2 Centre for Food Policy, School of Health and Medical Sciences, City St Georges, University of London, London, United Kingdom

**Keywords:** food environment, UK, food retail, fast food, food packaging, food safety, access to food, sustainable diets

## Abstract

Understanding food environments is crucial for developing policies and interventions to enhance the healthfulness and sustainability of UK diets. We systematically reviewed published scientific research to answer 2 research questions. First, what types and domains of the food environment have been assessed in the United Kingdom using what methodologies? Domains included availability, affordability, promotion, product characteristics/quality, convenience, and sustainability. Second, what outcomes have been assessed in relation to food environments? Outcomes were classified as descriptive (describing the food environment), dietary intake, and health. Articles published between January 2000 and December 2024 were identified by searching 7 databases: CAB Abstracts, CINAHL, EMBASE, Global Health, PubMed, Scopus, and Web of Science. A total of 31,457 articles were identified, 3418 full texts were reviewed, and 286 articles were included. Another 26 articles were included after screening the references of articles identified in the database search. Thus, data were extracted from a total of 312 articles. The most common domain studied was availability (*n =* 100, 32%), followed by product characteristics/quality (*n =* 94, 30%) and promotion (*n =* 33, 10%). There was a paucity of research on the domains of sustainability (*n =* 19, 6%) and affordability (*n =* 16, 5%), with no articles on the domain of convenience. Only 49 articles (16%) evaluated >1 domain. Most articles were descriptive (*n =* 206, 66%); 64 (20%) evaluated the association of the food environment with dietary intake and 42 (13%) evaluated the association with health, nearly all with obesity. The current literature on the food environment in the United Kingdom focuses largely on availability in the food retail space. More research is needed to understand how different domains of the food environment interact to influence dietary intake and health.

The protocol was registered at PROSPERO as CRD42022306066 on 8 February 2022.

## Introduction

Obesity has surpassed smoking as the leading contributor to death since 2014 in the United Kingdom [1]. The prevalence of obesity across the United Kingdom is high, with 32% of adults in Scotland having obesity [2], and 22% and 26% of adults in Wales [3] and England [4], respectively. By 2035, the prevalence of obesity in adults is predicted to increase by 5 percentage points in Scotland, 8 percentage points in England, and 11 percentage points in Wales [5]. Similarly worrying trends have been observed in children. From 2019 to 2020 to 2020 to 2021, the prevalence of obesity in children aged 4–5 y increased from 9.9% to 14.4% and in children aged 10–11 y, it increased from 21.0% to 25.5% [6]. Unhealthy diets underlie these worrying trends in obesity. The latest National Diet and Nutrition Survey (2023) found that consumption of fruits and vegetables is well below the 5-A-Day recommendation and mean intake of free sugars exceeds the maximum recommendation in all age groups [7].

Although many continue to place the onus of change on individuals, it is increasingly recognized that food environments that encourage the consumption of unhealthy foods are critical drivers of food choice [8]. The food environment is the interface between people and the wider food system. It encompasses all places where people access food, including retailers, restaurants, pubs/bars, cafes/coffee shops, takeaways, mobile food vans, schools, universities, workplaces, and charities as well as deliveries from these places [9]. The UK food environment has mirrored trends in unhealthy diets and obesity, with most evidence derived from the built environment. From 1980 to 2000, a study in North East England found a 79% increase in the total number of food outlets with a particularly marked increase in “foods for consumption away from home” outlets, which increased by 259% compared with a 16% increase in “household shopping” outlets [10]. Similar increases in availability of takeaways and grocers/convenience stores were reported around secondary schools in East London from 2001 to 2005 [11]. In 2022, there were an estimated 42,341 fast-food outlets across the United Kingdom [12]. Today, particularly after the COVID-19 pandemic, the way in which people in the United Kingdom procure food has diversified, with an increasing number of people ordering food online and using delivery services (e.g., Just Eat, Deliveroo, and Uber Eats) [13]. According to the Food Standards Agency’s “Food and You 2” survey of 5812 UK participants, conducted between April and July 2024, 75% of respondents reported shopping at large supermarkets, whereas 19% said that they used delivery applications such as Just Eat, Deliveroo, or Uber Eats at least once a week [14]. When asked about their preferences for ordering food or drinks online, 60% of respondents reported that they preferred to order from the websites of a restaurant, takeaway, or café.

To date, there has not been a comprehensive review of the literature on UK food environments. Previous, multicountry or United States-specific reviews do exist, however, and have focused on the retail food environment [[15], [16], [17]] or specific population subgroups, such as school children [[18], [19], [20]], or specific health outcomes, such as obesity [[21], [22], [23]]. There is also some recent interest in understanding the digital food environment given the widespread use of grocery and food delivery services in the United Kingdom, but this remains a largely unexplored area of research [24,25].

The aim of this systematic review was to identify and narratively summarize recent evidence regarding the UK food environment and to identify research gaps. The first research question was “what types and domains of food environments have been assessed using which methodologies?” The second was “which outcomes have been assessed in relation to food environments, including descriptive (describing the food environment), dietary intake, and health”. Furthermore, “how these outcomes have been stratified by area deprivation, education, gender, income, ethnicity, and age.” For all research questions, we explored how the number of articles differed by geography (e.g., UK-wide compared with England, Scotland, Wales, or Northern Ireland).

This systematic review provides an evidence-based understanding of food environment research in the United Kingdom, identifying geographic disparities and research gaps, and highlighting a need for bridging various food environment domains to foster cohesive changes and ultimately create healthier and more sustainable food systems.

## Methods

The protocol was registered with PROSPERO (ID: CRD42022306066) on 8 February 2022. Because this was not deemed human subjects’ research, it was exempt from institutional ethics committee review.

### Framework and definitions

The review was grounded in the Downs et al. [26] 2020 framework wherein 6 domains of food environments are defined, including availability, affordability, promotion, product characteristics/quality, convenience, and sustainability (Table 1) [26]. Although this framework proposes 3 types of food environments—built, cultivated, and natural—in the context of the United Kingdom, the built food environment is predominant [26].

**TABLE 1:** Six domains of food environments as proposed by Downs et al. (2020) [26].

Domain	Definition
Availability	The presence of a particular food item in a specific physical space or range
Affordability	The cost of food items in comparison to other foods or to income benchmarks (e.g., % of median income or % of poverty line)
Promotion	Factors that impact on the attractiveness of foods like packaging, labeling (including traffic light labeling) and placement in the store
Product characteristics (quality)	Features such as food packaging, nutrient and microbial content of foods, processing of foods and freshness of foods
Convenience	Time spent procuring, cooking and consuming foods
Sustainability	The environmental and social impact of food consumption

### Search strategy

The search strategy was developed by reviewing protocols on the food environment published in PROSPERO. Seven electronic databases were searched from inception through December 2024: CAB Abstracts, CINAHL, EMBASE, Global Health, PubMed, Scopus, and Web of Science. Searches included keywords for domains of the food environment (e.g., “food access∗” “supermarket” etc.) AND keywords for the geographic area of interest (e.g., “United Kingdom” “UK” etc.). The search terms and results for each database are given in Supplemental Table 1. Searches were duplicated by a second reviewer to check for accuracy. Additional articles were identified after reviewing the references of articles meeting inclusion criteria.

### Study selection

The eligibility criteria were as follows: research articles that measured ≥1 domain of the food environment (availability, affordability, promotion, product characteristics/quality, convenience, or sustainability); conducted in the United Kingdom (England, Wales, Scotland, or Northern Ireland); original research using quantitative or mixed methods with no restrictions on study design; and published from 2000 to December 2024 in English. Only studies published since 2000 were included to better inform local decision making (policymakers prioritize recent evidence) and subsequent research to address gaps in our understanding of UK food environments. The exclusion criteria were articles on food choices, personal factors such as taste, cultural preferences, knowledge about food, dietary intakes or behaviors without measuring food environments; qualitative articles; articles published in a language other than English; narrative reviews, systematic reviews, opinions, editorials, commentaries, or letters not reporting original research; and articles conducted outside the United Kingdom. If the research was conducted outside the United Kingdom but measured food environments in the United Kingdom, it was included. Articles on the home food environment were excluded. These included articles on marketing such as the impact of television advertising or time spent on television viewing in the home/personal food environment. This review only included articles on advertising in the built food environment – i.e., in-store promotions, packaging of foods, etc.

**FIGURE 1:**
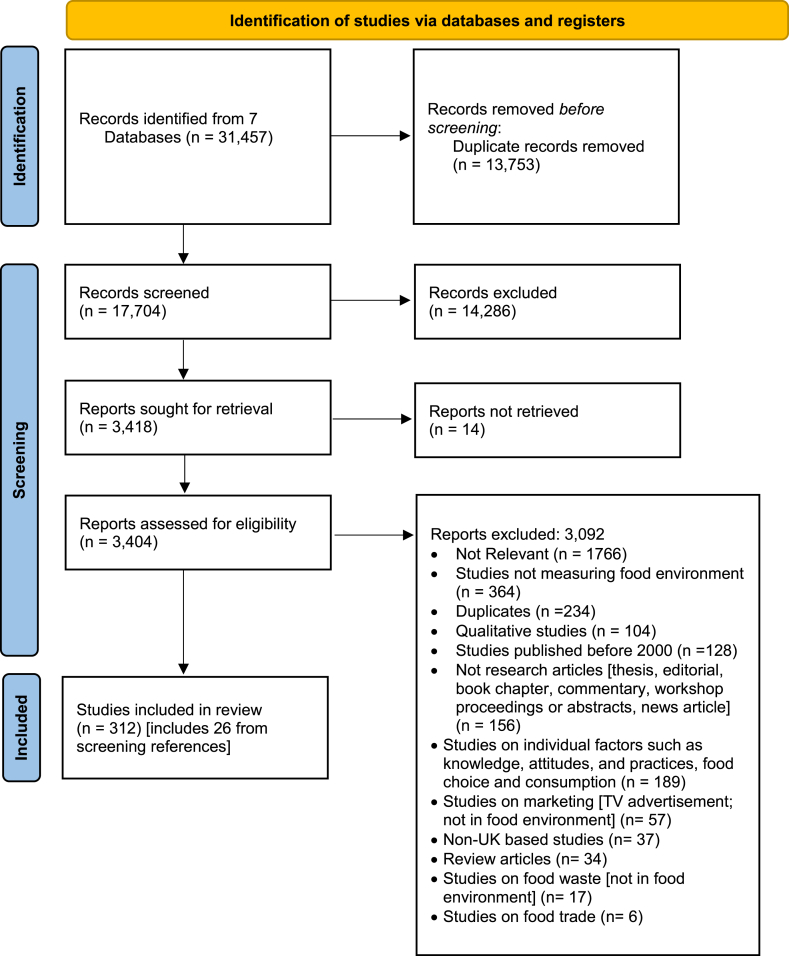
PRISMA flow diagram for systematic review of food environments in the United Kingdom.

Search results were imported into Covidence systematic review software (Veritas Health Innovation) for screening. The search yielded 31,457 articles, of which 13,753 were duplicates (Figure 1). DK and MDV independently screened titles and abstracts for eligibility, resulting in the exclusion of 14,286 records. Any discrepancies were resolved through discussion with LMJ. Interrater reliability was assessed using percent agreement (94.2%) and Cohen’s kappa (κ = 0.83), indicating substantial agreement between reviewers. Full texts of 3418 articles were sought for retrieval, of which full text of 14 articles was not available. The full texts of 3404 articles were then reviewed by DK and MVD. Of these, 3092 were excluded and 286 were included. Another 26 were included after screening the references of these 286 articles. Thus, the total number of articles included was 312.

### Data extraction

Data from all eligible articles were extracted into an Excel database. The Excel database was developed by DK with input from LMJ and tested on a subset of included articles, making iterative revisions to the database as necessary. DK and MVD extracted data, with uncertainties discussed and resolved with LMJ. Data were extracted on:

• Article characteristics. This included the last name of the first author, year of publication, year of data collection, country study was conducted in (UK-wide, England, Wales, Scotland, or Northern Ireland), study design, sample population, sample size, and source of funding.
• Type of food environment evaluated. Lytle and Sokol’s [27] categorization of the food environment was adapted to define the following 7 types of built food environments: *1*) food store environment (including grocery stores, supermarkets, convenience stores, snack bars, specialty food stores, and farmers’ markets); *2*) school food environment (including cafeterias, vending machines, and snack shops in day care settings, schools, colleges, and universities and the areas around them); *3*) worksite food environment (including cafeterias, vending machines, and snack shops in worksites); *4*) neighborhood food environment (all places to procure food within a physical region outside residential address); *5*) macro food environment (national and regional food supply); *6*) public facility food environment (including cafeterias, vending machines, and snack shops in recreation centers, healthcare facilities, and other public venues); and *7*) restaurant food environment.
• Domains of food environment evaluated (Table 1). This included availability, affordability, promotion, product characteristics/quality, convenience, and sustainability [26]. For the purposes of this systematic review, articles on food choices, personal factors such as taste, cultural preferences, and knowledge about food were not considered part of the food environment.
• Methodology used to assess the domains of the food environment. Any methodology was considered acceptable, including but not limited to instruments such as checklists, interviews or questionnaires; geographic analysis; sales data, nutrient and menu analysis. Lytle and Sokol’s [27] methodologies and instruments were adapted to define 12 types of methodologies, detailed in Table 2 [[28], [29], [30], [31], [32], [33], [34], [35], [36], [37], [38]]. For intervention studies conducted in the food environments, details on type of intervention were extracted.

**TABLE 2:** Definitions of methodologies to measure the food environment.

Name	Definition	Example
Geographic analysis	Analysis of data collected for a specific geographic area. This includes, e.g., counts of the number of food stores or restaurants; and distance to the nearest food stores or restaurants	Number of fast-food restaurants and convenience stores around home and school neighborhoods for 3089 adolescents [28]
Menu analysis	Collects standardized information from menus	Energy and nutritional content of menu items from 100 restaurants in the United Kingdom [29]
Market basket survey	Collects standardized information (on food characteristics, price, product placement, availability or including pictures of products) for a predefined list of foods via direct observation of the food environment or online. These foods may be based on foods frequently consumed by the population or foods of public health concern. Typically used in food store environments	Using a healthy food basket to determine availability and pricing of key items from shops in 2 localities [30]
Sales/purchase analysis	Use data from sales, cashier receipts, and annotated receipts to assess food purchasing patterns	An experimental study to examine the effect on vegetarian sales by increasing the proportion of vegetarian options available in university cafeterias [31]
Nutrient fact panel analysis	The nutrient content of foods available in a food environment is analyzed using existing information provided on the product itself (e.g., nutrient fact panel or claims on labeling) or using a nutrient database	Comparison of the Nutrition Information Panel content, serving size and package size of children's ready-to-eat breakfast cereals in 5 countries [32]
Nutrient analysis	Food samples are collected from a food outlet and analyzed in a laboratory for specific nutrients	Trans-fatty acid content of 62 processed food (pizza, garlic bread, breakfast cereals, quiche, fat spreads, fish and meat products, chips, savory snacks, confectionery, and ice cream) purchased from supermarkets, independent retailers and takeaway outlets [33]
Contaminant analysis	Food samples are collected from a food outlet and analyzed in a laboratory for contaminants such as pesticides or pathogens	Assessment of the microbiological safety of salad vegetables and sauces from kebab takeaway restaurants in the United Kingdom [34]
Physical measurements	Data collected via physical measurements of stores such as aisle length, shelf length, and placement	Association of supermarket size (measured as total aisle length) and national obesity prevalence in England [35]
Ecologic footprint analysis	Life cycle assessments determine the environmental impact of foods available in food environments	Environmental Impact Score of sandwiches and beverages available in 18 university-owned food outlets [36]
Policy analysis	Articles analyzing policies or recommendations that impact on the domains of food environments such as taxes or food labeling requirements	Banning the promotion of foods high in fat, sugar, and salt in Scotland has the potential to reduce the number of calories, sugar, saturated fats, and sodium for most food groups [37]
Food supply analysis	Uses national-level data such as food prices, food availability, or food consumption	Modeling study to shift current diets to diets that meet dietary recommendations for health, have lower greenhouse gas emissions, and are affordable for different income groups [38]
• Outcome assessment. This included information on the type of outcome (descriptive, diet, or health), outcome assessment method, and any stratification by area deprivation, education, gender, income, ethnicity, and age.

Details on variables extracted from observational and intervention studies are listed in Supplemental Table 2. This systematic review assessed attributes such as the number of articles measuring the food environment across geographies (i.e., Wales, England, Scotland, Northern Ireland, and UK wide); the number of articles assessing the type of measure (e.g., geographic analysis, menu analysis, nutrient fact panel analysis, etc.); and the environment in which the measurement tool was used (e.g., food store, restaurant, school, etc.). No formal risk of bias assessment was done. Details for all included articles in the systematic review (*n =* 312) are listed in Supplemental Table 3.

## Results

**FIGURE 2:**
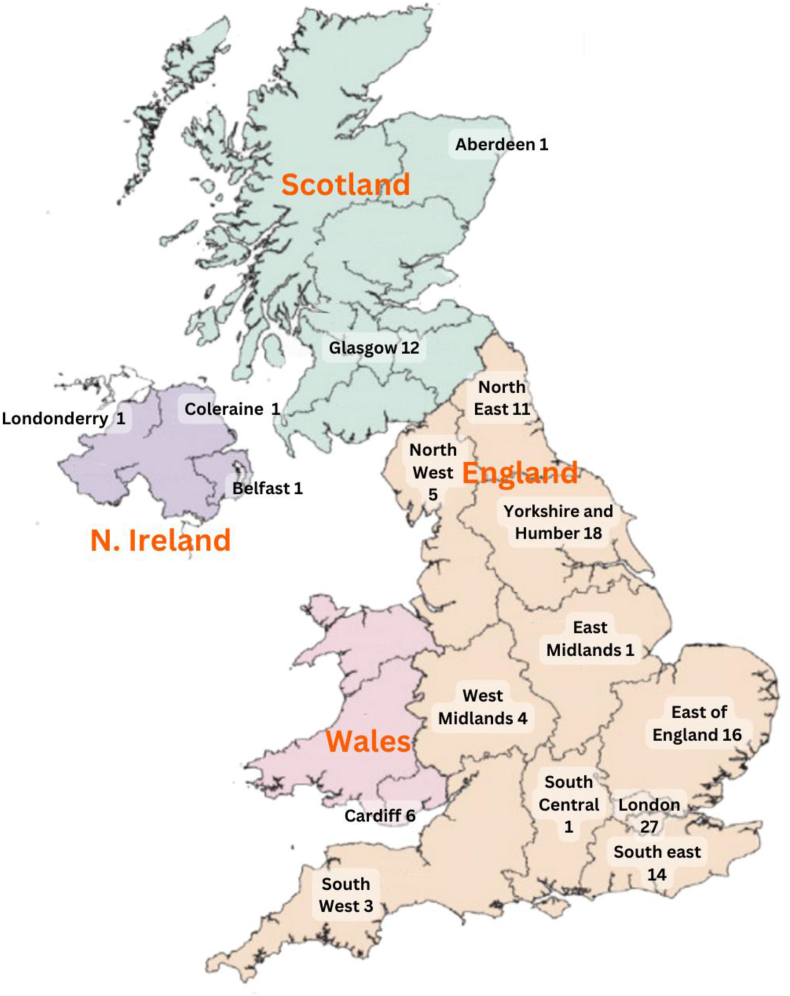
Geographic distribution of number of articles by country in a systematic review of the UK food environment (*n* = 312).

Key characteristics of articles included in the systematic review are presented in Table 3. Most articles were from England (*n =* 120, 38% [10,11,21,24,28,30,31,36,[38], [39], [40], [41], [42], [43], [44], [45], [46], [47], [48], [49], [50], [51], [52], [53], [54], [55], [56], [57], [58], [59], [60], [61], [62], [63], [64], [65], [66], [67], [68], [69], [70], [71], [72], [73], [74], [75], [76], [77], [78], [79], [80], [81], [82], [83], [84], [85], [86], [87], [88], [89], [90], [91], [92], [93], [94], [95], [96], [97], [98], [99], [100], [101], [102], [103], [104], [105], [106], [107], [108], [109], [110], [111], [112], [113], [114], [115], [116], [117], [118], [119], [120], [121], [122], [123], [124], [125], [126], [127], [128], [129], [130], [131], [132], [133], [134], [135], [136], [137], [138], [139], [140], [141], [142], [143], [144], [145], [146], [147], [148], [149], [150]]), followed by UK-wide articles (*n =* 87, 28%), Scotland (*n =* 27, 9% [37,[151], [152], [153], [154], [155], [156], [157], [158], [159], [160], [161], [162], [163], [164], [165], [166], [167], [168], [169], [170], [171], [172], [173], [174], [175], [176]]), Northern Ireland (*n =* 9, 3% [[177], [178], [179], [180], [181], [182], [183], [184], [185]]), and Wales (*n =* 10, 3% [[186], [187], [188], [189], [190], [191], [192], [193], [194], [195]]). There were 7 articles from Great Britain [[196], [197], [198], [199], [200], [201], [202]] and 21 (6%) multicountry studies [32,35,[203], [204], [205], [206], [207], [208], [209], [210], [211], [212], [213], [214], [215], [216], [217], [218], [219], [220], [221]]. We further categorized the number of articles at the regional level in each country, showing clear preferences and paucity of food environment research in some areas (Figure 2). In England, most articles were from London (*n =* 27, 26%) and Yorkshire and Humber (*n =* 18, 15%); in Scotland they were from Glasgow (*n =* 12, 43%) and in Wales from Cardiff (*n =* 6, 67%). Within each region, details of urban or rural areas were not provided. Only 19 articles evaluated urban/rural differences [53,56,61,84,99,104,114,133,138,145,162,164,169,173,174,177,181,185,197]. After the search and analysis of articles had been conducted, 1 article retraction was published [222].

**TABLE 3:** Key characteristics of articles included in the systematic review of the UK food environment (*n =* 312).

Characteristic	*n* (%) or range
Geography	
UK-wide	87 (28)
England	120 (38)
Scotland	27 (9)
Northern Ireland	9 (3)
Wales	10 (3)
Great Britain	7 (2)
Coastal waters of United Kingdom	1 (1)
Scotland and England	3 (1)
Multicountry	21 (7)
Not able to assign	27 (8)
Location	
Not specified	262 (84)
Both rural and urban	18 (6)
Only urban	32 (10)
Year of publication	
2000–2005	23 (7)
2006–2010	30 (10)
2011–2015	75 (24)
2016–2020	103 (33)
Beyond 2020	81 (26)
Year of data collection	
Not reported	76 (24)
≤2000	12 (4)
2001–2005	18 (6)
2006–2010	47 (15)
2011–2015	55 (18)
2016–2020	88 (28)
Beyond 2020	16 (5)
Population	
Infant	7 (2)
Children	35 (11)
Adolescents	26 (9)
Adults	57 (18)
Elderly	3 (1)
N/A	184 (59)
Study design	
Cross sectional	242 (78)
Longitudinal	31 (10)
Case study	10 (3)
Modeling	6 (2)
Randomized controlled trial	6 (2)
Intervention	17 (5)
Sample size	
People	115–42,838
Store	3–8864
Food samples or products	101–68,153
Meals	8–2,255,404
Areas	3–6781
Type of food(s) evaluated	
Unhealthy foods (fast foods, sweets, cakes, pastries, etc.)	26 (9)
Healthy foods (salads, whole grain cereals, dried fruits, nuts etc.)	11 (4)
Mix of healthy and unhealthy foods (salads and confectionery)	31 (10)
Fruits and vegetables	18 (6)
Meat and seafood	17 (5)
Milk and milk products	13 (4)
Beverages (including alcoholic beverages)	9 (3)
Bread	5 (2)
Baby/infant food	3 (1)
Articles on multiple food groups	9 (3)
Ready-to-eat	34 (11)
Special foods (low protein, gluten free, meat alternatives, etc.)	7 (2)
Meals (meals served at schools, restaurants, workplaces, etc.)	32 (10)
Food outlets	97 (31)
Source of funding	
Government	161 (52)
Charitable NGOs, foundations, or professional societies	33 (10)
Intergovermental bodies	9 (3)
Private charities	5 (2)
Joint funding (government and industry)	1 (0)
Joint funding (government and private charity)	1 (0)
Not mentioned	63 (20)
None received	39 (13)

Over the past decade, research on food environments has expanded significantly, with 58% of articles (*n =* 184) published after 2015 and 26% (*n =* 81) after 2020. However, only 5% (*n =* 16, [24,96,99,103,119,203,217,[223], [224], [225], [226], [227], [228], [229], [230], [231]]) of these articles noted data collection occurring after 2020. Most articles (*n =* 184, 59%) did not focus on any population group such as children or the elderly but on measuring food environment features. Most articles were cross sectional (*n =* 242, 78%), followed by longitudinal analysis (*n =* 31, 10% [10,11,52,56,72,81,83,86,96,99,108,145,166,192,211,222,227,228,[232], [233], [234], [235], [236], [237], [238], [239], [240], [241], [242], [243], [244]], intervention studies (*n =* 17, 5% [31,39,54,73,74,95,122,123,130,167,178,212,229,[245], [246], [247], [248]]), case studies (*n =* 10, 3% [42,78,160,[187], [188], [189],^191^,^249^,^250^]), and 2% each (*n =* 6) were randomized controlled trials [119,120,134,135,137,224] and modeling studies [68,140,201,[251], [252], [253]].

Because this review focused on multiple domains of the food environment, the sample size ranged from 115 to 42,838 people; 3 to 8864 stores; 101 to 68,153 food samples or products; 8 to 2,255,404 meals, and 3 to 6781 areas. On tabulation of articles based on type of food studied, 31% (*n =* 97) of the articles focused on type of food outlets instead of focusing on any particular food or food group [10,11,29,35,[42], [43], [44],^47^,[49], [50], [51], [52], [53],[56], [57], [58],[60], [61], [62], [63],^66^,^68^,^70^,^71^,[76], [77], [78],[81], [82], [83], [84], [85], [86], [87], [88],^91^,[100], [101], [102], [103], [104], [105], [106], [107], [108], [109], [110], [96], [97], [98], [99],[114], [115], [116],[125], [126], [127],^129^,^130^,^133^,^138^,[148], [149], [150],^152^,[155], [156], [157], [158],^162^,^164^,^169^,^175^,^183^,[188], [189], [190],^196^,^197^,^200^,^205^,^214^,^216^,^217^,^227^,^232^,^238^,^250^,[254], [255], [256], [257], [258], [259], [260], [261], [262], [263], [264], [265], [266]]. These were followed by articles on ready-to-eat foods (*n =* 34, 11% [32,74,92,111,117,121,122,142,146,165,166,176,178,[191], [192], [193],^195^,^198^,^210^,^215^,[267], [268], [269], [270], [271], [272], [273], [274], [275], [276], [277], [278], [279], [280]]), and articles on meals served at schools, restaurants, or workplaces (*n =* 32, 10% [31,39,46,54,69,73,79,80,93,94,106,[119], [120], [121],^134^,^136^,^139^,^147^,^167^,^187^,^209^,^224^,^228^,[281], [282], [283], [284], [285], [286], [287], [288]]). Of 312 articles, 210 (67%) stated their source of funding. Among these, 161 articles (52%) that received government funding, 33 (10%) articles were funded by charitable NGOs, foundations, or professional societies, 9 (3%) articles were funded by intergovermental bodies like WHO, and 5 articles (2%) received funding from private charities [83,116,122,126,289]. One article noted joint funding from government and industry [190], whereas another stated joint funding from government and a private charity [221]. A total of 63 articles (20%) did not mention their source of funding and 39 articles (12%) did not receive any funding.

### Types of food environments

**FIGURE 3:**
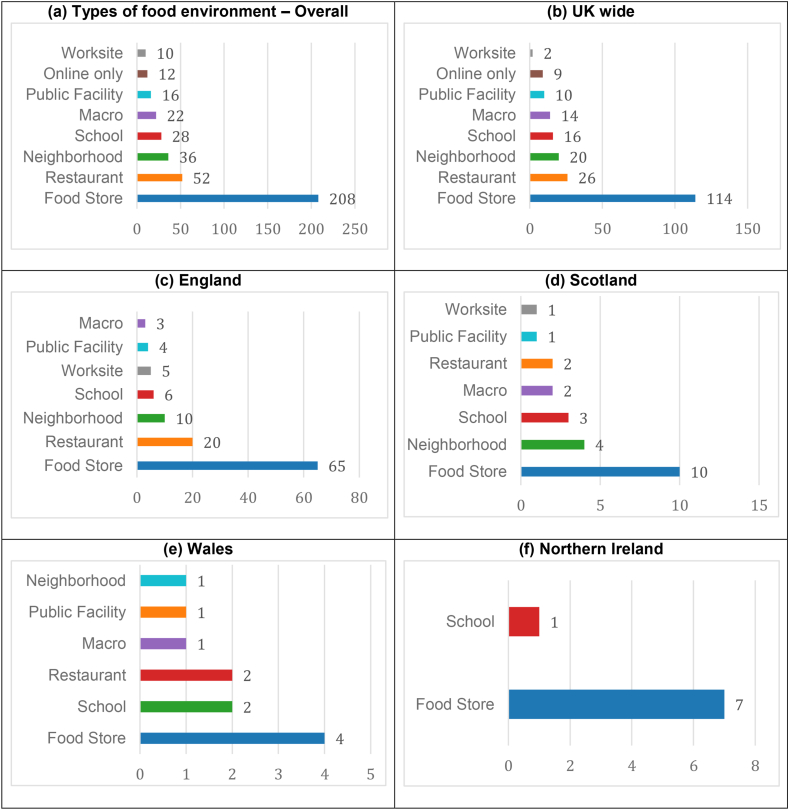
Number of articles by type of food environment and country in a systematic review of the UK food environment. Categories are nonexclusive, i.e., articles that evaluated >1 type of food environment are counted more than once.

Articles on food store environments were the most common (*n =* 208, 67% [41,46,61,67,72,82,[88], [89], [90],^115^,^122^,^140^,^142^,^148^,^150^,^151^,^166^,^168^,^184^,^196^,^201^,^205^,^206^,^213^,^219^,^222^,^229^,^230^,^234^,^235^,^241^,^244^,^246^,^248^,[253], [254], [255],^260^,^265^,^277^,^281^,[290], [291], [292], [293], [294], [295], [296]]) (Figure 3). These included articles on the nutrient content [40,45,64,124,131,146,147,172,198,210,211,218,220,236,240,242,267,269,270,[272], [273], [274],^278^,^280^,^288^,^289^,[297], [298], [299], [300], [301], [302], [303], [304], [305], [306], [307]] and microbial content [102,170,174,[178], [179], [180],^182^,^207^,^208^,^212^,^308^] of foods sold in UK food stores, availability of healthy foods [51,70,137,153,154,163,181,188,189,233,239,246,309] and access to food stores [37,48,49,60,68,77,114,118,144,145,149,157,159,160,177,197,200,260,310]. The next most prevalent food environment was restaurant food environments (*n =* 52, 16% [39,54,62,98,101,110,132,158,217,237,250,257,268,311]), which included articles on nutrient content [29,80,93,119,126,209,216,224,227,261,263,264,266,282,284] or microbiological quality [34,111,283,[312], [313], [314]] of meals served at fast food or full service restaurants. Thirty-six articles evaluated different aspects of neighborhood [38,10,42,50,59,91,103,127,128,143,256,315] such as 20-min neighborhood [169] or out of home access in deprived neighborhoods [66,87,95,104,108,109,133,138,155,164,173,188,199,251,258,316]. Articles assessing nutritional content of school meals [36,79,139,187,247,317] or vending machines [117] at schools were categorized under school food environments (*n =* 28, 9% [11,28,43,78,81,84,119,125,129,141,152,156,183,193,214,245,286]). Twelve articles assessed the online food environment: 8 UK-wide articles [25,223,225,226,231,249,318], 3 from England [55,96,99], and 1 multicountry study [204]. There were 15 articles on hospitals and other public venues categorized as public facility food environment [69,71,74,76,92,112,113,121,130,136,175,191,194,285,319]; 8 articles on worksite food environment [73,94,107,119,134,135,167,228] and 19 articles on macro food environment assessing impact of food policies [53,63,165,171,215,221,238,252,259,279,292,[320], [321], [322], [323], [324], [325], [326], [327]]. There were 22 (7%) articles that evaluated 2 types of food environments [44,56,58,82,186,195,271], of which 13 were on food store and restaurant food environment [57,86,87,97,113,192,262,328]. For example, articles evaluating microbial quality of food samples collected from food stores and fast-food restaurants [111,329].

There were no articles on natural food environments (both wild and cultivated). All of the above were classified as the built food environment.

### Domains and methodologies

**FIGURE 4:**
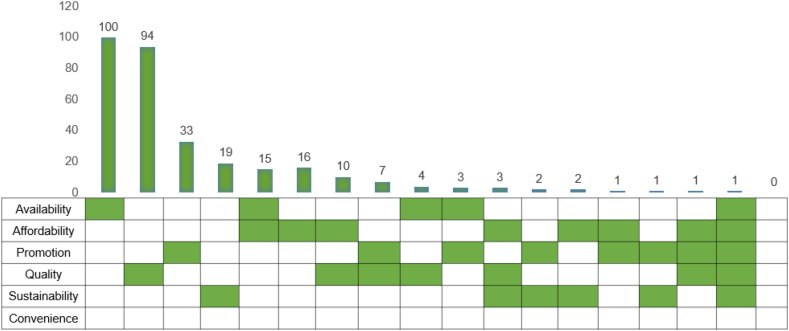
Number of articles by domain of food environment in a systematic review of the UK food environment (*n* = 312). The colored boxes represent the domains, whereas the number on the bar represents the number of articles in the domains. The presence of multiple, colored boxes signifies >1 domain.

The most common domain studied was availability (*n =* 100, 32% [10,11,24,28,35,[42], [43], [44],^49^,^50^,^52^,^53^,[56], [57], [58], [59], [60], [61], [62], [63],^66^,^68^,^71^,[76], [77], [78],^81^,^82^,[84], [85], [86], [87], [88],[90], [91], [92],[95], [96], [97], [98], [99], [100], [101],^103^,^104^,[108], [109], [110],^114^,^116^,[119], [120], [121],^125^,^127^,^129^,^133^,^138^,^140^,^131^,[143], [144], [145],^149^,^150^,^152^,[154], [155], [156], [157], [158],^161^,^162^,^169^,^173^,^181^,^183^,[188], [189], [190],^197^,^199^,^200^,^214^,^217^,^232^,^238^,^246^,^254^,[256], [257], [258], [259], [260],^279^,^309^,^311^,^316^,^330^,^331^]), followed closely by product characteristics/quality (*n =* 94, 30% [29,[32], [33], [34],^40^,^45^,^64^,^67^,^69^,^80^,^93^,^94^,^102^,^106^,[111], [112], [113],^115^,^117^,^128^,^131^,^136^,^146^,^151^,^170^,^174^,^176^,[178], [179], [180],^182^,^184^,[191], [192], [193], [194], [195],^198^,[207], [208], [209], [210], [211], [212],^216^,^220^,^223^,^227^,^236^,^240^,^242^,^261^,^263^,^264^,^266^,^267^,[269], [270], [271], [272], [273], [274],[276], [277], [278],[280], [281], [282], [283], [284], [285], [286],^288^,^289^,^293^,[297], [298], [299], [300], [301], [302], [303], [304],^308^,[313], [314], [315],^318^,^329^,[332], [333], [334]]) and promotion (*n =* 33, 10% [25,72,75,83,90,122,132,134,135,137,148,165,166,168,175,186,196,203,205,215,219,230,231,[244], [245], [246], [247], [248],^253^,^265^,^275^,^294^,^335^]) (Figure 4). There was a paucity of research on the domains of sustainability (*n =* 19, 6% [31,54,79,206,228,[249], [250], [251], [252],^255^,^268^,^287^,^292^,^320^,^322^,^323^,^325^,^336^,^337^]) and affordability (*n =* 16, 5% [37,48,89,118,171,185,201,213,234,235,237,239,241,290,310,324]). There were no articles on the domain of convenience. Under the domain availability, most articles focused on assessing density or proximity of food outlets [52,232]. Others focused on the type of foods available in food stores [95,120,130]. These included fresh fruits and vegetables, and ready-to-eat and unhealthy foods (e.g., soft drinks, chips, confectionery, etc.). Under the domain product characteristics/quality, most articles assessed nutrient content (e.g., fatty acids, trans-fat, sodium, sugar, etc.) [146,242,276] or microbial pathogens in food store or restaurant food environments [34,314]. Articles on marketing and nutritional claims on food packaging were covered under the promotion domain [203,275,335], whereas those on food prices were most common under the affordability domain [201,321]. Finally, articles on the environmental impact of food were covered under sustainability [336].

There were 50 articles (16%) that evaluated >1 domain, most common were articles evaluating availability and affordability (*n =* 15, [30,48,51,55,65,70,107,153,163,164,167,177,188,190,262]), and articles assessing affordability and product characteristics/quality (*n =* 10, [41,47,48,147,224,226,233,291,321,326]). One article evaluated all domains except convenience. It was an 11-country study to benchmark the implementation of recommended nutrition policies by national governments using the Healthy Food Environment Policy Index [221]. The most studied domain in England, Scotland, and Northern Ireland was availability, whereas in Wales and UK-wide articles it was quality. More details can be found in Supplemental Table 3.

**FIGURE 5:**
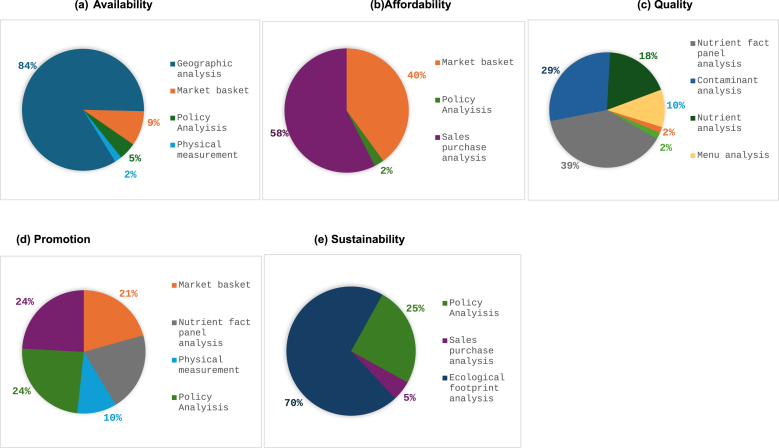
Type of methodology under each domain of food environment in a systematic review of the UK food environment. Categories are nonexclusive, i.e., articles that used >1 methodology are counted more than once.

There was a clear preferred methodology to measure each domain (Figure 5). However, because several articles assessed multiple domains, the categories are not mutually exclusive and therefore have been counted more than once. Geographic analysis was the most common methodology used to assess availability, applied in 84% (*n =* 108) of articles on availability [311]. This was followed by market basket surveys (*n =* 10, 9%) [162] policy analysis (*n =* 5, 5%) [125] and physical measurements (*n =* 2, 2%) [35]. Assessing food purchase patterns using sales/cashier receipts (*n =* 23, 58%) [47], market basket surveys (*n =* 16, 40%) [48], and policy analysis (*n =* 1, 2%) [221] were the most used methodologies to assess the affordability domain. To measure promotion, 7 (24%) articles used sales/purchase analysis [75] and policy analysis [145] each, 6 articles (21%) used nutrient information available on the package called nutrient fact panel analysis [335] and market basket surveys [83] each, and 3 articles (10%) used physical measurements [173]. Nutrient fact panel analysis (*n =* 44, 39%) [32], contaminant analysis (*n =* 33, 29%) [332], articles on food samples tested in a laboratory, called nutrient analysis (*n =* 21,18%) [94], menu analysis (*n =* 12, 10%) [29], market basket surveys [204], and policy analysis (*n =* 2, 2%) [286] each were methodologies to evaluate the domain on product characteristics/quality.

Finally, to measure sustainability, ecologic footprint analysis (*n =* 14, 70%), policy analysis (*n =* 5, 25%) [336], and sales/purchase analysis (*n =* 1, 5%) [228] were used. It is important to note that within ecologic footprint analysis, multiple methodologies were used, such as life cycle analysis [287,320], reduction in livestock product supply [252], and Water Footprint Impact Indicator estimated as scarcity weighted liters per portion and global hectares per annum [36]. This highlights the multifaceted nature of sustainability definitions and data sources.

### Outcomes

Overall, most articles (*n =* 206, 66%) were descriptive and did not assess any associations between the food environment, 64 (20%) assessed associations with dietary intake [11,33,39,44,45,60,69,80,[93], [94], [95],^107^,^115^,^117^,^120^,^124^,^126^,^128^,^132^,^134^,^135^,^139^,^143^,^146^,^147^,^152^,^176^,^216^,^227^,^233^,^234^,^236^,^239^,^240^,^242^,^246^,^264^,^266^,^267^,[269], [270], [271], [272], [273], [274], [275], [276], [277], [278],^280^,^281^,^285^,^288^,[297], [298], [299],^301^,^302^,^304^,^306^,^315^,^318^,^326^,^335^], and 42 (13%) articles assessed associations with health [35,50,56,58,59,[61], [62], [63],^66^,^76^,^77^,^81^,^82^,[84], [85], [86],8[9], [10], [11], [12], [13], [14], [15], [16], [17], [18], [19], [20], [21], [22], [23], [24], [25], [26], [27], [28], [29], [30], [31], [32], [33], [34], [35], [36], [37], [38], [39], [40], [41], [42], [43], [44], [45], [46], [47], [48], [49], [50], [51], [52], [53], [54], [55], [56], [57], [58], [59], [60], [61], [62], [63], [64], [65], [66], [67], [68], [69], [70], [71], [72], [73], [74], [75], [76], [77], [78], [79], [80], [81], [82], [83], [84], [85], [86], [87], [88], [89], [90], [91],^103^,^106^,^110^,^133^,^140^,^141^,^144^,^145^,^149^,^200^,^201^,^223^,^232^,^238^,^251^,^256^,^258^,^259^,^279^,^311^,^316^,^323^] (Table 4). Of the articles assessing health associations, all focused on obesity and the impact of food outlet proximity or density on BMI, except 4 articles: 1 analyzed links with type 2 diabetes [279], 2 focused on cardiovascular disease and cancer [252,258], and another with type 2 diabetes, cardiovascular disease, and cancer [251]. No country-wise differences were observed: descriptive articles were most common across all countries (Table 4).

**TABLE 4:** Outcomes stated in articles included in the systematic review of the UK food environment (*n =* 312).

Country	Outcomes *n* (%)		
	None descriptive	Diet	Health
UK wide	108 (54)	50 (79)	24 (58)
England	48 (23)	12 (18)	17 (40)
Scotland	26 (13)	2 (3)	0 (0)
Wales	9 (4)	0 (0)	0 (0)
Northern Ireland	9 (4)	0 (0)	0 (0)
Multiple countries within United Kingdom	6 (3)	0 (0)	1 (2)
Total	206	64	42

Most of the articles with outcomes did not present stratified analyses (*n =* 233, 75%); 64 (20%) articles did stratified analysis by a single variable [30,37,38,43,48,49,51,55,66,70,71,77,78,[83], [84], [85], [86], [87],^91^,^95^,^96^,^98^,^99^,^101^,^104^,^108^,^114^,^122^,^125^,^138^,^144^,^145^,^149^,^154^,[156], [157], [158],[161], [162], [163], [164], [165],^168^,^169^,^172^,^173^,^177^,[188], [189], [190],^196^,^198^,^199^,^217^,^235^,^238^,^246^,^247^,^254^,^256^,^257^,^316^,^30^,^330^] and 15 (5%) articles conducted stratified analysis using 2 or more variables [44,53,82,90,98,109,129,133,140,141,229,258,259,292,322] (Supplemental Table 4). Area deprivation was the most common variable for stratification, for example, articles comparing food outlet density in the least and most deprived neighborhoods [48,96].

## Discussion

A comprehensive understanding of the UK food environment requires interdisciplinary research involving public health experts, nutritionists, behavioral scientists, geographers, and complex systems scientists, among others. However, this systematic review found that most research to date has involved only one or a few aspects of the food environment. Although >250 articles have been published on the UK food environment over the past 2 decades, most were on a single domain (availability) and in a single type of food environment, food store. Moreover, obesity was the only health outcome studied extensively. Given recent diversification of the way in which people in the United Kingdom procure food, with an increasing number of people ordering food and using delivery services [13], and the cost-of-living crisis, climate crisis, EU exit, and other disruptions to the UK food supply, more interdisciplinary work is needed to explore how interactions across multiple domains impact dietary intake and health.

Furthermore, despite evidence that convenience is a key driver of food consumption behavior in the United Kingdom [13] as well as the impact of food systems on climate change [338], this systematic review identified little research on these food environment domains: convenience and sustainability. According to the FAO, the processing, packaging, and transport of food have overtaken agriculture as the largest contributor to food-related greenhouse gas emissions in many high-income countries [339]. Similarly, evidence suggests that time spent on home food preparation is an indicator of healthy diets [340] and lack of time is a leading barrier to adopting dietary recommendations [341], yet there were no articles identified under the domain of convenience. Consumer interest in sustainability and convenience are megatrends of the fast-food sector in the United Kingdom, evident with nearly half of UK adults buying more locally sourced food and expecting food businesses to play a role in climate change [12]. There is also a continued high demand for home food delivery after pandemic [12].

Only approximately one-fifth of articles identified in this review evaluated >1 domain of the food environment. Valuable insights have come from the few articles identified in this review that looked at multiple domains. For example, the Healthy Food Environment Policy Index, which aimed to assess the extent of implementation of recommended food environment policies by governments, provided a holistic view of the United Kingdom’s food environment [221]. It also identified priority actions to meet implementation gaps [221]. Another article evaluating multiple domains identified in this review looked at what dietary changes are required to shift the UK population to diets that meet dietary recommendations for health, have lower greenhouse gas emissions, and are affordable for different income groups [38]. To fully comprehend the impact of the food environment on human and planetary health, research is needed that evaluates multiple domains and how these domains interact with each other to influence food choice. For example, ready-to-eat foods are convenient, but are often less affordable, less healthy, come in plastic packaging, and require refrigeration, which impacts their sustainability [342].

With regard to the type of food environment studied, food store environments have been the most researched food environment type by far. More than half of the articles (67%) in this review were on the food store environment, followed by restaurants (16%), neighborhood food environments (11%), and school food environments (9%). These findings differ slightly from Lytle and Sokol’s [27] systematic review of articles measuring the food environment published between 2007 and 2015, which found that 73% of articles measured the food store environment, 50% measured restaurants, and 15% measured schools (percent do not add to 100 because some articles measured both). The emphasis on food store environments is appropriate given that 71% of expenditures on food and nonalcoholic drinks in the United Kingdom is at stores (with the remaining 29% of expenditures eaten out) [343]. However, there is an increasing need to evaluate the online food environments given the rise in takeaways and deliveries [344], supermarket home delivery, and other forms of home delivery (e.g., vegetable boxes, Hello Fresh, and Amazon Fresh) [13]. We found only 7 articles (2%) that assessed the online food environment in this systematic review, focusing on either availability or labeling of food items in the retail food environment.

Approximately 70% of articles identified in this review were descriptive with no association with dietary intake or health outcomes. Among the few articles that evaluated associations with health outcomes, all but 4 evaluated the association with obesity. The other 4 studied type 2 diabetes, cardiovascular disease, and cancer. This is expected because obesity is the leading risk factor for mortality in the United Kingdom [1], but other diet-related diseases such as type 2 diabetes, hypertension, and heart disease should also be explored.

This systematic review is not without limitations. First, we did not include search terms for food banks or charity shops, which are an increasingly important source of food during the cost-of-living crisis [345]. We also did not include search terms explicitly related to cultivated or natural food environments (e.g., community gardens), and therefore may have missed literature on these types of food environments. Second, grey literature such as third sector or government reports may have been missed. We tried to overcome this by searching 7 databases and reviewing the reference list for all included articles but cannot guarantee that a relevant report was not missed. Third, the search terms used for “convenience” may have contributed to the lack of studies identified for this domain. Future work should consider expanding the search terms and definition to include the time cost of preparing and consuming food as well as personal motivation to plan/prepare meals, availability of ingredients and cooking equipment in the home, and access to transport to procure ingredients.

This study advances understanding of the knowledge gaps that must be filled to design evidence-based policies to improve the healthfulness and sustainability of UK diets. At the same time, there is enough evidence for governments to act to improve local food environments to achieve healthy diet and weight goals [346]. A recently published review of systematic reviews on the effectiveness of food environment policies in improving population diets found that food environment policies targeting the availability of foods in retail and food establishments, food provision in school settings, product reformulation, and the size of portions/packages are effective [347]. There are many recent examples of the United Kingdom and devolved government actions to improve the food environment. For example, the ban of single-use plastics in England that was initiated from October 2023 [348] and initiatives to reduce food waste [349,350] have the potential to improve the sustainability of food environments. Regulations on the promotion of foods and beverages high in fat, sugar, and salt in England [351] and under consideration in Scotland [352] and Wales [353] have the potential to improve the healthfulness of food environments across the United Kingdom. A data visualization tool has also been developed to help local authorities explore their food environments [354]. There is a need for a comprehensive review of policies across the United Kingdom, including nonfood policies and monitoring of the impact of these policies on dietary intake, health, and food environments. The better we understand the food environment, the easier it will be to create interventions that bring about a positive change in public health and planetary health.

To summarize, the current literature on the food environment in the United Kingdom focuses almost exclusively on availability in the food retail space. Although several recent government initiatives aim to improve the healthfulness of food environments in the United Kingdom, more research is needed to understand how different domains of the food environment interact to influence dietary intake and health. Moreover, the types of food environments evaluated need to be expanded to include the increasingly relevant digital food environment.

## Author contributions

The authors’ responsibilities were as follows – LMJ, DK: conceived and designed the study; DK, MDVM: screened and extracted data; LMJ, DK: contributed to the development of the extraction template; DK: performed the analysis and drafted the manuscript; DK: has primary responsibility for the final content; all authors: read and approved of the final manuscript.

## Data availability

Extracted data are publicly and freely available without restriction at https://figshare.com/articles/dataset/_b_The_UK_food_environment_a_systematic_review_of_domains_methodologies_and_outcomes_b_/29374151?file=55544885.

## Funding

CR is funded by the UK Food Systems Centre for Doctoral Training, The Partnership for Sustainable Food Future Centre for Doctoral Training (Project Reference: BB/V011391/1. CR is funded by the Healthy soil, Healthy food, Healthy people (H3) project (Project Reference: BB/V004719/1). These programs are funded through the Strategic Priorities Fund (SPF) “Transforming the UK Food System for Healthy People and a Healthy Environment Programme” delivered by UKRI, in partnership with the Global Food Security Programme, BBSRC, ESRC, MRC, NERC, Defra, DHSC, PHE, Innovate UK, and FSA. CR is also funded by the Co-Centre for Sustainable Food Systems (Project Reference: BB/Y012909/1) and Joined up Landscapes (Project Reference: TBA).

## Conflict of interest

CR has advisory positions on boards at the Nutrition Society (Food systems theme lead) and the Institute of Food Science & Technology (Sustainability working group). CR is part of the Sustainable Diet Working Group, Faculty of Public Health, and the British Standards Institution/International Organization for Standardization committee ISO/TC 34/SC 20 (Food loss and waste). CR has received payment via City, University of London for consulting for: WRAP (a UK NGO); Zero Waste Scotland; DEFRA and the FSA (UK government). CR has been paid a Speaker’s Stipend by the following events: The Folger Institute (2020). CR is a member of EGEA, and Nutrition Society Scientific Committees, and co-chair of a session of the EGEA conference (2023) and Nutrition Society conferences (2022–2025). This has meant that his registration and flight/accommodation have been paid by Aprifel or the Nutrition Society. CR has won competitive research funding (€49,858) from the following independent foundation: The Alpro Foundation (2020). All other authors report no conflicts of interest.
