# Supplementary material for: The UK Food Environment: A Systematic Review of Domains, Methodologies, and Outcomes
Source: Curr Dev Nutr. 2025 Oct 11;9(11):107573. doi: 10.1016/j.cdnut.2025.107573 (PMC12685522; doi:10.1016/j.cdnut.2025.107573)
Supplement: Multimedia component 1 [file mmc1.docx]

**The UK food environment: a systematic review of domains, methodologies and outcomes**

Deksha Kapoor Kirsteen Shields, Christian Reynolds, Lindsay M. Jaacks

**Online Supplementary Material**

**Table of Contents:**

| **Supplementary Material** | **Page Number** |
| --- | --- |
| Supplementary Table 1: Search strategies used for each database | 2-4 |
| Supplementary Table 2: Variables extracted from all included articles in the systematic review (n=312) | 5-6 |
| Supplementary Table 3: Country wise distribution of food environment domains | 7 |
| Supplementary Table 4: Outcomes (with stratification) | 8 |

| **Supplementary Table 1: Search strategy used for each database** | | |
| --- | --- | --- |
| Database | Search strategy | Records Identified on October 2024 |
| Pubmed | ("sustainable diet*" OR "Food Access*" OR "nutrition label*" OR "Food Label*" OR "Menu Label*" OR "Food Desirability" OR "Food Market*" OR "Food Advertising" OR "food pric*" OR "food suppl*" OR "Food outlet*" OR "Convenience Store*" OR "supermarket*" OR "food retail*" OR "food store*" OR "grocery store*" OR "fast food*" OR "Food Kiosk*" OR "Vending Machine*" OR "Point of Purchase" OR "Restaurant*" OR "Canteen*" OR "Café*" OR "Food Swamp*" OR "Food Desert*" OR "Food Milieu*" OR "takeaway*" OR "take?away" OR " take away" OR "corner store" OR " grocery store" OR "food store" OR "food retail" OR supermarket OR "convenience store" OR "food outlet" OR "Food Sustainability" OR "Food Convenience" OR "food quality" OR "food promot*" OR "food afford*" OR "food cost*" OR "food avail*" OR Foodscape OR " obesogenic environment" OR "nutrition environment" OR "food environment") AND ("Scottish"[Tiab] OR "British"[Tiab] OR "English"[Tiab] OR "Welsh"[Tiab] OR "Great Britain"[Tiab] OR "GB"[Tiab] OR "Northern Ireland"[Tiab] OR "England"[Tiab] OR "Wales"[Tiab] OR "United Kingdom"[Tiab] OR "UK"[Tiab] OR "Scotland"[Tiab]) | 3402 |
| CAB Abstracts | (("sustainable diet*" OR "Food Access*" OR "nutrition label*" OR "Food Label*" OR "Menu Label*" OR "Food Desirability" OR "Food Market*" OR "Food Advertising" OR "food pric*" OR "food suppl*" OR "Food outlet*" OR "Convenience Store*" OR "supermarket*" OR "food retail*" OR "food store*" OR "grocery store*" OR "fast food*" OR "Food Kiosk*" OR "Vending Machine*" OR "Point of Purchase" OR "Restaurant*" OR "Canteen*" OR "Café*" OR "Food Swamp*" OR "Food Desert*" OR "Food Milieu*" OR "take away" OR "corner store" OR " grocery store" OR "food store" OR "food retail" OR supermarket OR "convenience store" OR "food outlet" OR "Food Sustainability" OR "Food Convenience" OR "food quality" OR "food promot*" OR "food afford*" OR "food cost*" OR "food avail*" OR Foodscape OR "obesogenic environment" OR "nutrition environment" OR "food environment") AND ("Scottish" OR "British" OR "English" OR "Welsh" OR "Great Britain" OR "GB" OR "Northern Ireland" OR "England" OR "Wales" OR "United Kingdom" OR "UK" OR "Scotland")) AND ( ((language:(( "English" ) )) geographic-location: “UK" )) | 3920 |
| Global Health | (ab:("Scottish" OR "British" OR "English" OR "Welsh" OR "Great Britain" OR "GB" OR "Northern Ireland" OR "England" OR "Wales" OR "United Kingdom" OR "UK" OR "Scotland") AND ("sustainable diet*" OR "Food Access*" OR "nutrition label*" OR "Food Label*" OR "Menu Label*" OR "Food Desirability" OR "Food Market*" OR "Food Advertising" OR "food pric*" OR "food suppl*" OR "Food outlet*" OR "Convenience Store*" OR "supermarket*" OR "food retail*" OR "food store*" OR "grocery store*" OR "fast food*" OR "Food Kiosk*" OR "Vending Machine*" OR "Point of Purchase" OR "Restaurant*" OR "Canteen*" OR "Café*" OR "Food Swamp*" OR "Food Desert*" OR "Food Milieu*" OR "take away" OR "corner store" OR " grocery store" OR "food store" OR "food retail" OR supermarket OR "convenience store" OR "food outlet" OR "Food Sustainability" OR "Food Convenience" OR "food quality" OR "food promot*" OR "food afford*" OR "food cost*" OR "food avail*" OR Foodscape OR "obesogenic environment" OR "nutrition environment" OR "food environment" )) AND (geographic-location:(( "UK" OR "England" ) )) (language:(( "English" ) )) )) | 4122 |
| Web of Science | (AB=("Scottish" OR "British" OR "English" OR "Welsh" OR "Great Britain" OR "GB" OR "Northern Ireland" OR "England" OR "Wales" OR "United Kingdom" OR "UK" OR "Scotland")) AND ALL=("sustainable diet*" OR "Food Access*" OR "nutrition label*" OR "Food Label*" OR "Menu Label*" OR "Food Desirability" OR "Food Market*" OR "Food Advertising" OR "food pric*" OR "food suppl*" OR "Food outlet*" OR "Convenience Store*" OR "supermarket*" OR "food retail*" OR "food store*" OR "grocery store*" OR "fast food*" OR "Food Kiosk*" OR "Vending Machine*" OR "Point of Purchase" OR "Restaurant*" OR "Canteen*" OR "Café*" OR "Food Swamp*" OR "Food Desert*" OR "Food Milieu*" OR "take away" OR "corner store" OR " grocery store" OR "food store" OR "food retail" OR supermarket OR "convenience store" OR "food outlet" OR "Food Sustainability" OR "Food Convenience" OR "food quality" OR "food promot*" OR "food afford*" OR "food cost*" OR "food avail*" OR foodscapes OR "obesogenic environment" OR "nutrition environment" OR "food environment" ) | 9062 |
| CINAHL | AB ( "Scottish" OR "British" OR "English" OR "Welsh" OR "Great Britain" OR "GB" OR "Northern Ireland" OR "England" OR "Wales" OR "United Kingdom" OR "UK" OR "Scotland" ) AND TX ( "sustainable diet*" OR "Food Access*" OR "nutrition label*" OR "Food Label*" OR "Menu Label*" OR "Food Desirability" OR "Food Market*" OR "Food Advertising" OR "food pric*" OR "food suppl*" OR "Food outlet*" OR "Convenience Store*" OR "supermarket*" OR "food retail*" OR "food store*" OR "grocery store*" OR "fast food*" OR "Food Kiosk*" OR "Vending Machine*" OR "Point of Purchase" OR "Restaurant*" OR "Canteen*" OR "Café*" OR "Food Swamp*" OR "Food Desert*" OR "Food Milieu*" OR "take away" OR "corner store" OR " grocery store" OR "food store" OR "food retail" OR supermarket OR "convenience store" OR "food outlet" OR "Food Sustainability" OR "Food Convenience" OR "food quality" OR "food promot*" OR "food afford*" OR "food cost*" OR "food avail*" OR Foodscape OR "obesogenic environment" OR "nutrition environment" OR "food environment" ) | 966 |
| EMBASE | ("sustainable diet*" or "Food Access*" or "nutrition label*" or "Food Label*" or "Menu Label*" or "Food Desirability" or "Food Market*" or "Food Advertising" or "food pric*" or "food suppl*" or "Food outlet*" or "Convenience Store*" or "supermarket*" or "food retail*" or "food store*" or "grocery store*" or "fast food*" or "Food Kiosk*" or "Vending Machine*" or "Point of Purchase" or "Restaurant*" or "Canteen*" or "Café*" or "Food Swamp*" or "Food Desert*" or "Food Milieu*" or "take away" or "corner store" or " grocery store" or "food store" or "food retail" or supermarket or "convenience store" or "food outlet" or "Food Sustainability" or "Food Convenience" or "food quality" or "food promot*" or "food afford*" or "food cost*" or "food avail*" or Foodscape or "obesogenic environment" or "nutrition environment" or "food environment").af. and ("Scottish" or "British" or "English" or "Welsh" or "Great Britain" or "GB" or "Northern Ireland" or "England" or "Wales" or "United Kingdom" or "UK" or "Scotland").ab. | 4952 |
| Scopus | ( TITLE-ABS-KEY ( "sustainable diet*"  OR  "Food Access*"  OR  "nutrition label*"  OR  "Food Label*"  OR  "Menu Label*"  OR  "Food Desirability"  OR  "Food Market*"  OR  "Food Advertising"  OR  "food pric*"  OR  "food suppl*"  OR  "Food outlet*"  OR  "Convenience Store*"  OR  "supermarket*"  OR  "food retail*"  OR  "food store*"  OR  "grocery store*"  OR  "fast food*"  OR  "Food Kiosk*"  OR  "Vending Machine*"  OR  "Point of Purchase"  OR  "Restaurant*"  OR  "Canteen*"  OR  "Café*"  OR  "Food Swamp*"  OR  "Food Desert*"  OR  "Food Milieu*"  OR  "take away"  OR  "corner store"  OR  " grocery store"  OR  "food store"  OR  "food retail"  OR  "convenience store"  OR  "food outlet"  OR  "Food Sustainability"  OR  "Food Convenience"  OR  "food quality"  OR  "food promot*"  OR  "food afford*"  OR  "food cost*"  OR  "food avail*"  OR  "Foodscape"  OR  "obesogenic environment"  OR  "nutrition environment"  OR  "food environment" )  AND  TITLE-ABS-KEY ( "Scottish"  OR  "British"  OR  "English"  OR  "Welsh"  OR  "Great Britain"  OR  "GB"  OR  "Northern Ireland"  OR  "England"  OR  "Wales"  OR  "United Kingdom"  OR  "UK"  OR  "Scotland" ) )  AND  ( LIMIT-TO ( AFFILCOUNTRY ,  "United Kingdom" ) )  AND  ( LIMIT-TO ( LANGUAGE ,  "English" ) ) | 5033 |

| **Supplementary Table 2:** Variables extracted from included articles in the systematic review (n=312) | | | |
| --- | --- | --- | --- |
| S. No | Variable Name | Details | Referred in manuscript |
| 1 | Author Name | Name of the first author | Not included in results, used for data management |
| 2 | Year | Year of Publication | Table 3 |
| 3 | Country | England, Scotland, Wales, Northern Ireland or UK | Table 3 and Figure 2 |
| 4 | Region | Name of area or region within the country if mentioned | Figure 2 |
| 5 | International Territorial Levels (ITLs) classification | ITL is a geocode standard for referencing the administrative divisions of countries for statistical purposes. |  |
| 6 | Location | Urban, rural, N/A | Table 3 |
| 7 | Year(s) of Study | Year of data collection | Table 3 |
| 8 | Population | Adults, School Children, Adolescents, Infants Elderly, N/A | Table 3 |
| 9 | Study Design | E.g., Cross-sectional, Case study, Intervention etc. | Table 3 |
| 10 | Sample size | Sample size reported | Reported in text (lines 187-189) |
| 11 | Type of Food Environment | Food Store Environment, Macro Food Environment, Public Facility Food Environment, Restaurant Food Environment, School Food Environment, Neighbourhood Food Environment and Worksite Food Environment | Figure 3 |
| 12 | Domain of Food Environment | Availability, Affordability, Promotion, Quality, Convenience, Sustainability | Figure 4 |
| 13 | Food(s) Evaluated | E.g., fruits and vegetables, breakfast cereals etc. | Table 3 |
| 14 | Methodologies to Measure | Geographic analysis, Food supply analysis,  Menu analysis, Market basket survey,  Sales/ purchase analysis, Nutrient fact panel analysis, Nutrient analysis, Contaminant analysis, Physical measurements, Ecological footprint analysis, Policy analysis  For intervention studies, details on type of intervention were extracted. | Figure 5 |
| 15 | Outcome | None-Descriptive, Diet, Health | Table 4 |
| 16 | Stratification (in analysis) | Area Deprivation, Education, Gender, Income, None | Supplementary table 5 |
| 17 | Result | Short summary of result | Not included for this narrative summary |
| 18 | Funding Sources | Details on funding for the research | Table 3 |

| **Supplementary Table 3: Country wise distribution of food environment domains*** | | | | | | |
| --- | --- | --- | --- | --- | --- | --- |
|  | Domain | UK wide | England | Scotland | N. Ireland | Wales |
| Single domains | Availability | 51 | 37 | 12 | 2 | 2 |
|  | Affordability | 10 | 3 | 2 |  |  |
|  | Promotion | 21 | 7 | 4 |  |  |
|  | Product characteristics (‘Quality’) | 73 | 13 | 5 | 4 | 5 |
|  | Sustainability | 18 | 3 | 1 | 1 |  |
| Double domains | Affordability, Promotion |  |  | 1 |  |  |
|  | Affordability, Quality | 9 |  |  |  |  |
|  | Availability, Quality |  | 2 | 1 |  |  |
|  | Availability, Affordability | 6 | 5 | 3 | 1 |  |
|  | Availability, Promotion | 1 | 1 |  |  | 1 |
|  | Promotion, Quality | 8 |  |  |  |  |
|  | Promotion, Sustainability | 1 |  |  |  |  |
|  | Sustainability, Affordability | 1 |  | 1 |  |  |
|  | Sustainability, Quality | 1 | 1 |  |  |  |
| 3 domains | Affordability, Promotion, Quality | 1 |  |  |  |  |
|  | Affordability, Quality, Sustainability | 3 |  |  |  |  |
| 5 domains | All domains except convenience |  | 1 |  |  |  |

* *Categories are non-exclusive, i.e., studies that were conducted in multiple countries are counted more than once*

| **Supplementary Table 4: Outcomes (with stratification)** | | | | | |
| --- | --- | --- | --- | --- | --- |
| Stratification | | None-Descriptive | Diet | Health | Total |
| None | None | 173 | 59 | 1 | 233 |
| Single variable | Area Deprivation | 45 | 4 |  | 49 |
|  | Gender | 1 |  |  | 1 |
|  | Age | 1 |  |  | 1 |
|  | Income | 11 |  |  | 11 |
|  | Education | 2 |  |  | 2 |
| 2  variables | Area Deprivation, Education |  | 1 |  | 1 |
|  | Area Deprivation, Gender | 1 |  |  | 1 |
|  | Area Deprivation, Income | 5 |  |  | 5 |
|  | Area Deprivation, Ethnicity | 2 |  |  | 2 |
|  | Gender, Education | 1 |  |  | 1 |
|  | Gender, Age | 1 |  |  | 1 |
| Multiple variables | Area Deprivation, Gender, Ethnicity, Education |  |  | 1 | 1 |
|  | Gender, Ethnicity, Income | 1 |  |  | 1 |
|  | Area Deprivation, Gender, Income, Ethnicity, Education Level, Employment, Age, | 1 |  |  | 1 |
|  | Area Deprivation, Income, Education | 1 |  |  | 1 |
|  | Total | 246 | 64 | 2 | 312 |
